# In-depth comparative transcriptome analysis of intestines of red swamp crayfish, *Procambarus clarkii*, infected with WSSV

**DOI:** 10.1038/srep26780

**Published:** 2016-06-10

**Authors:** Zhiqiang Du, Yanhui Jin, Daming Ren

**Affiliations:** 1 School of life science and technology, Inner Mongolia University of Science and Technology, Baotou, Inner Mongolia autonomous region 014010, China; 2 College of Biological Science and Technology, Shenyang Agriculture University, Shenyang, Liaoning 110866, China

## Abstract

Crayfish has become one of the most important farmed aquatic species in China due to its excellent disease resistance against bacteria and viruses. However, the antiviral mechanism of crayfish is still not very clear. In the present study, many high-quality sequence reads from crayfish intestine were obtained using Illumina-based transcriptome sequencing. For the normal group (GN), 44,600,142 high-quality clean reads were randomly assembled to produce 125,394 contigs. For the WSSV-challenged group (GW), 47,790,746 high-quality clean reads were randomly assembled to produce 148,983 contigs. After GO annotation, 39,482 unigenes were annotated into three ontologies: biological processes, cellular components, and molecular functions. In addition, 15,959 unigenes were mapped to 25 different COG categories. Moreover, 7,000 DEGs were screened out after a comparative analysis between the GN and GW samples, which were mapped into 250 KEGG pathways. Among these pathways, 36 were obviously changed (P-values < 0.05) and 28 pathways were extremely significantly changed (P-values < 0.01). Finally, five key DEGs involved in the JAK-STAT signaling pathway were chosen for qRT-PCR. The results showed that these five DEGs were obviously up-regulated at 36 h post WSSV infection in crayfish intestine. These results provide new insight into crayfish antiviral immunity mechanisms.

Invertebrates lack an acquired immune system but develop an innate immune system to defend against pathogenic microorganisms. The innate immune system mainly comprises cellular and humoral immune responses and contains an enormous number of innate immune-related genes^1^. When hosts suffer a challenge or infection due to a pathogen, these genes can be synergistically mobilized to play their respective roles in defense, especially in the humoral immune response^2^. It is crucial to study the function of immune-related genes to completely determine the coordination mechanisms of the innate immune system.

Red swamp crayfish is usually used as a model organism to research the response principles of the invertebrate innate immune system. This species is native to northeastern Mexico and South America and was introduced to China from Japan in the 1930s^3^. Because of its good characteristics of fitness, strong adaptability to changing environment, and high fecundity, red swamp crayfish has been widely cultured in China^4^. Currently, this species has become one of the most economically important farmed aquatic species due to its excellent disease resistance against bacteria, fungi, and viruses. Studies of red swamp crayfish have revealed detailed antibacterial and antifungal mechanisms, such as the Toll pathway and the Imd pathway, among others^5^; however, antiviral mechanisms remain unclear^6^. Thus, it is necessary to screen for antiviral genes and antivirus-related signaling pathways through transcriptome sequencing.

Recently, there have been some reports of the transcriptome sequencing of crayfish tissues such as eyestalk, hepatopancreas, muscle, ovary, testis, spermary, epidermis, branchia, and stomach. Transcriptome data for crayfish intestine and WSSV-challenged tissues have not been reported. More importantly, the invertebrate intestinal innate immune response is a crucial defense mechanism against external pathogens. The intestinal tract is a complex ecosystem containing a diverse pathogenic community^7^. The intestine is an important organ that can remove invading pathogens via an efficient and specific immune pattern^8^. The study of the intestinal transcriptomes is an important part of the research of the innate immune response mechanism.

In recent years, next-generation sequencing (NGS) technology has been widely used to screen out large amounts of genetic information in model organisms^9^. NGS technology is superior in many aspects to the traditional Sanger sequencing technology. NGS technology can provide enormous amounts of sequence data in a much shorter amount of time and at a much cheaper cost^10^. The expressed sequences produced using NGS technology are usually ten-fold or one-hundred-fold greater than those that are produced using traditional Sanger sequencing technology^11^. In the present study, Hi-Seq technology was used to sequence the intestinal transcriptomes of crayfish from a normal group (GN) and a WSSV-challenged group (GW). This information was used to generate expression profiles and to discover differentially expressed genes between normal crayfish and WSSV-challenged crayfish. Moreover, the functions of differently expressed genes (DEGs) were annotated and classified by the Gene Ontology (GO) database, Clusters of Orthologous Groups (COG) database, and Kyoto Encyclopedia of Genes and Genomes (KEGG) database. These data provide an important resource for research on the gene functions, molecular events, and signaling pathways relating to the invertebrate antivirus immune response.

## Results and Discussion

### Illumina sequencing of the crayfish intestinal transcriptome

Illumina-based RNA sequencing was carried out with two sets of crayfish intestine samples (GN and GW). After cleaning and quality testing the GN sample, a total of 44,600,142 clean reads were screened out from 46,945,132 raw reads, corresponding to 4,460,014,200 total nucleotides (nt). The Q20 percentage (percentage of bases whose quality was greater than 20 in clean reads), N percentage (percentage of uncertain bases after filtering), and GC percentage were 98.05%, 0.00% and 40.45%, respectively (Table 1). For the GW sample, a total of 47,790,746 clean reads were screened out from 49,574,674 raw reads, corresponding to 4,779,074,600 nt. The Q20 percentage, N percentage, and GC percentage were 97.96%, 0.00% and 41.58%, respectively (Table 1). All of these sequences were used for further analysis.

### *De novo* assembly of the transcriptome

**Figure 1:**
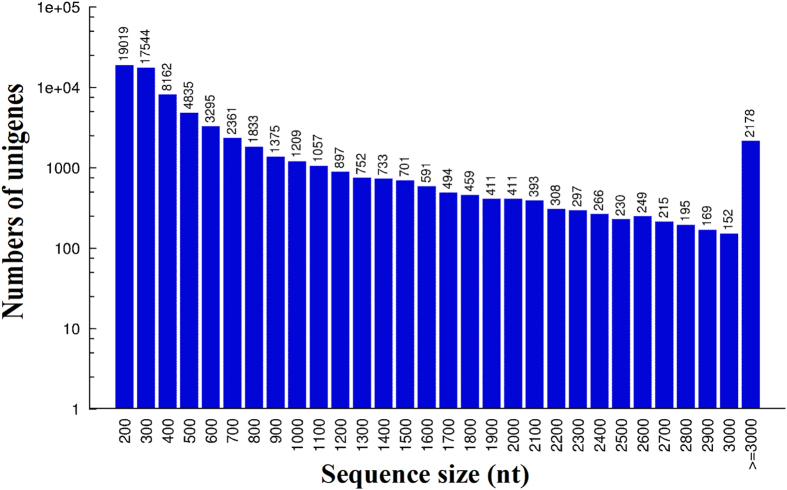
Distribution of the length of all of the assembled unigenes in normal crayfish intestine (GN).

**Figure 2:**
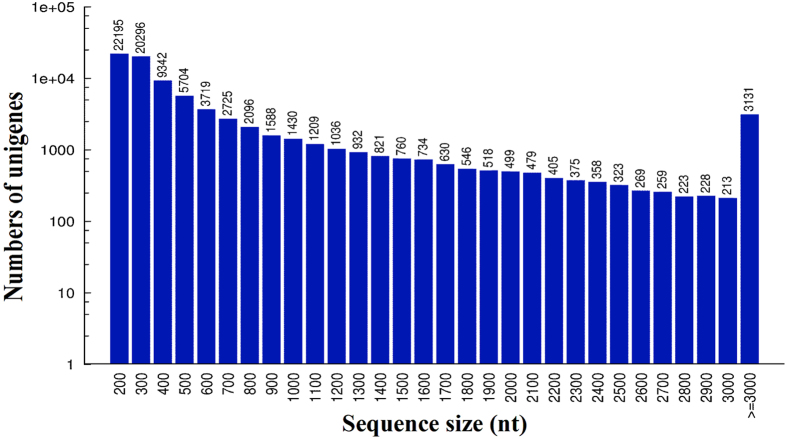
Distribution of the length of all of the assembled unigenes in WSSV-challenged crayfish intestine (GW).

After low-quality reads and short reads were removed, high-quality clean reads were used to carry out transcriptome *de novo* assembly using Trinity software with the default parameters^12^. For the GN sample, a total of 44,600,142 high-quality clean reads were randomly assembled to produce 125,394 contigs with an N50 of 701 bp. The contigs were further assembled and clustered into 70,791 unigenes with a mean length of 627 nt that were composed of 7,352 distinct clusters and 63,439 distinct singletons. In addition, the N50 of the above unigenes was 1,258 bp (Table 2). For the GW sample, a total of 47,790,746 high-quality clean reads were randomly assembled to produce 148,983 contigs with an N50 of 769 bp. The contigs were further assembled and clustered into 83,043 unigenes with a mean length of 672 nt that were composed of 9,686 distinct clusters and 73,357 distinct singletons. In addition, the N50 of the above unigenes was 1,456 bp (Table 2). The distributions of the unigene sequence lengths for the GN and GW samples are shown in Figs 1 and 2, respectively.

### Functional annotation of predicted proteins

After transcriptome *de novo* assembly for two sets of crayfish intestine samples (GN and GW), the transcripts were used for annotation, in combination with previously reported data from two other transcriptomes^13^. First, sequence annotation was carried out based on unigenes from the merged group^14^. Then, the putative functions of all of the unigenes were analyzed based on GO and COG classifications^15^. In this study, before the analysis of DEGs associated with white spot syndrome virus (WSSV) infection, a basic sequence analysis of the merged group transcriptome data (98,676 unigenes) was performed to understand functions of the crayfish intestine transcriptome. Among the predictable sequences, a total of 39,482 unigene sequences were annotated using BLASTx alignment with an E-value ≤ 10E-5. A total of 35,539 (90.01%), 14,931 (37.82%), 28,221 (71.48%), 25,290 (64.05%), 15,595 (39.50%), and 13,848 (35.07%) unigenes had significant matches with sequences in the non-redundant sequence (Nr), non-redundant nucleotide (Nt), Swiss-Prot, KEGG, COG and GO databases, respectively. In brief, a total of 35,539 transcripts (90.01% of all of the annotated transcripts) had significant hits in the Nr protein database. The gene names of the top BLAST hits were assigned to each transcript with significant hits. Among these transcripts, 2,682 (7.55%) were matched with genes from *Paramecium tetraurelia*, 2,362 (6.65%) were matched with genes from *Daphnia pulex*, 3,265 (9.19%) were matched with genes from *Tetrahymena thermophila*, 1,302 (3.66%) were matched with genes from *Tribolium castaneum*, 1,292 (3.64%) were matched with genes from *Ichthyophthirius multifiliis*, and 929 (2.61%) were matched with genes from *Pediculus humanus*.

In addition, a GO classification analysis was carried out; GO classification is an internationally standardized gene function classification system. This analysis provides a dynamically updated controlled vocabulary and can exactly define gene characteristics and products in any organism^16^. This analysis includes three ontologies: biological process, cellular component, and molecular function^17^. The biological process ontology includes biological adhesion, biological regulation, cell killing, cellular component organization or biogenesis, cellular process, developmental process, establishment of localization, growth, immune system process, localization, locomotion, metabolic process, multi-organism process, multicellular organismal process, negative regulation of biological process, negative regulation of biological process, positive regulation of biological process, regulation of biological process, reproduction, reproductive process, response to stimulus, rhythmic process, signaling, and single-organism process. The cellular component ontology includes cell, cell junction, cell part, extracellular matrix, extracellular matrix part, extracellular region, extracellular region part, macromolecular complex, membrane, membrane part, membrane-enclosed lumen, organelle, organelle part, synapse, synapse part, virion, and virion part. The molecular function ontology includes antioxidant activity, binding, catalytic activity, electron carrier activity, enzyme regulator activity, molecular transducer activity, nucleic acid binding transcription factor activity, protein binding transcription factor activity, receptor activity, structural molecule activity, and transporter activity.

**Figure 3:**
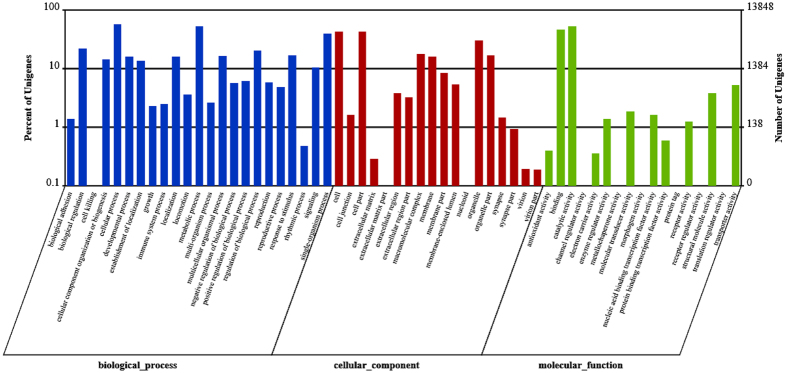
Gene ontology (GO) classification of transcripts of two intestine sample (GN and GW) sets. The three main GO categories include biological process, cellular component, and molecular function.

In the present study, a GO analysis was carried out using Blast2GO software. A total of 13,848 transcripts that were annotated in the GO database were categorized into 58 functional groups, including three main GO ontologies: biological processes, cellular components, and molecular functions (Fig. 3). Among these functional groups, the terms “biological regulation”, “cellular process”, “metabolic process”, “cell”, “single-organism process”, “cell part”, “binding”, and “catalytic activity” were dominant.

**Figure 4:**
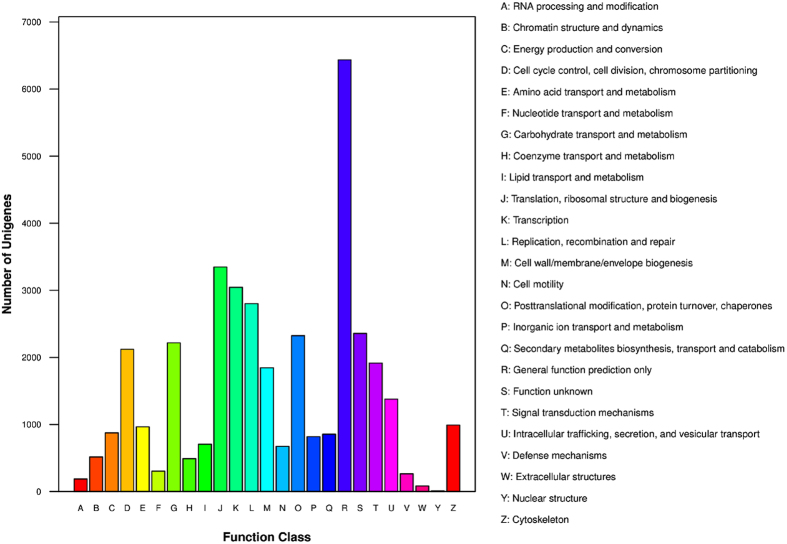
Cluster of orthologous groups (COG) classification of putative proteins.

COGs were delineated by comparing protein sequences encoded in the complete genome, representing major phylogenetic lineages^18^. In this study, COG classification was used to further evaluate the completeness of the transcriptome library and the effectiveness of annotation methods. As a result, a total of 15,959 unigenes were mapped into 25 different COG categories. Of these categories, the largest COG group was the R category, representing “general function prediction only” (6,435 unigenes, 41.26%); followed by the J category, representing “translation, ribosomal structure and biogenesis” (3,347 unigenes, 21.46%); the K category, representing “transcription” (3,044 unigenes, 19.52%); and the L category, representing “replication, recombination and repair” (2,802 unigenes, 17.97%) (Fig. 4).

In addition, KEGG is a bioinformatics resource for linking genomes to life and the environment^19^. The KEGG PATHWAY database records networks of molecular interactions in the cells and variants specific to particular organisms^20^. The genes from the merged groups (GN and GW) were categorized using the KEGG database to obtain more information to predict unigene function^21^. As a result, a total of 25,290 unigenes were classified into 257 KEGG pathways. Among these KEGG pathways, the top 50 statistically significant KEGG classifications are shown in Table 3. Some important innate immunity-related pathways were predicted in this KEGG database, including *Vibrio* cholerae infection (1092 sequences, 4.32%), focal adhesion (910 sequences, 3.6%), Epstein-Barr virus infection (860 sequences, 3.40%), lysosome (610 sequences, 2.41%), HTLV-I infection (596 sequences, 2.36%), Herpes simplex infection (593 sequences, 2.34%), salmonella infection (576 sequences, 2.28%), MAPK signaling pathway (542 sequences, 2.14%), adherens junction (467 sequences, 1.85%), and so on (Table 3). It is worth noting that the insulin signaling pathway, the Wnt signaling pathway, the mRNA surveillance pathway, endocytosis, phagosome, ECM-receptor interaction, bacterial invasion of epithelial cells, Fc gamma R-mediated phagocytosis, and tight junction were present in the top 50 statistically significant KEGG classification. Perhaps these signaling pathways and related genes have an important effect on further understanding the antiviral mechanisms of the innate immune system.

### Differentially expressed gene analysis in crayfish intestine after WSSV infection

**Figure 5:**
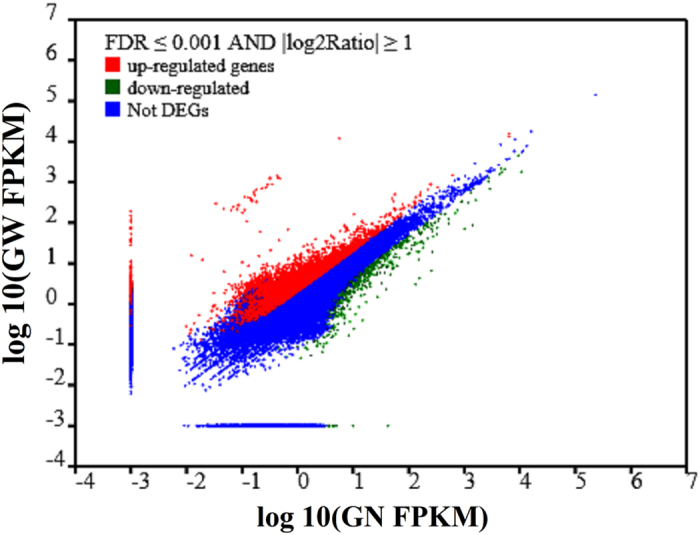
Comparative analysis of gene expression levels for two transcript libraries between the normal crayfish intestine (GN) and WSSV-challenged crayfish intestine (GW) samples. Red dots represent transcripts that were significantly up-regulated in GW, and green dots indicate that those transcripts were significantly down-regulated. The parameters “FDR ≤ 0.001” and “|log2 Ratio| ≥ 1” were used as the thresholds to judge the significance of gene expression differences.

Previous sequence analysis and annotation for all of the unigenes in the merged group (GN and GW) provided some valuable information to analyze the crayfish intestine transcriptome. However, the variation in the gene expression level of crayfish intestine after WSSV infection was expected. In this study, FDR ≤ 0.001 and an absolute value of log2Ratio ≥ 1 were used as the filtering thresholds to identify up-regulated or down-regulated genes between normal crayfish and WSSV-challenged crayfish. As shown in Fig. 5, a total of 7,000 DEGs were screened out after a comparative analysis between the GN and GW samples. Among these genes, 5,976 were identified as differentially up-regulated and 1,024 as differently down-regulated by more than two fold. Among these 7,000 DEGs, 6,821 genes existed in both the GN and GW samples at the same time, including 5,798 differentially up-regulated genes and 1,024 differently down-regulated genes. Moreover, 178 genes were only found in the GW sample after WSSV challenge. Compared to the abovementioned signaling pathways, these 178 genes have an important influence on further studies of the antiviral immune mechanisms.

**Figure 6:**
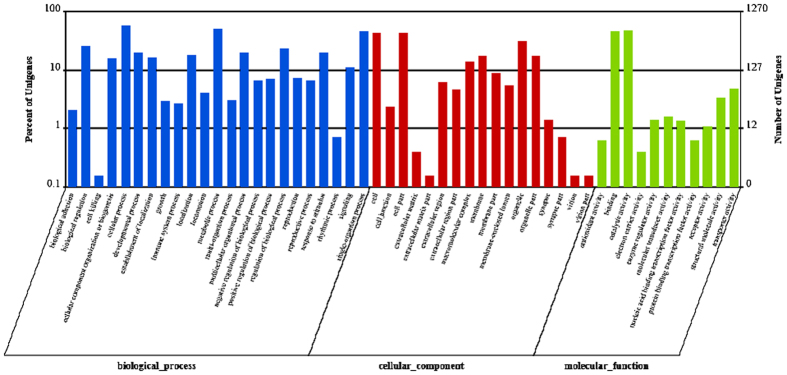
Gene ontology (GO) classification analysis of DEGs between the GN and GW samples. The three main GO categories included biological process, cellular component, and molecular function.

To determine the biological function of DEGs between GN and GW, GO classification and KEGG pathway analysis were carried out^22^. GO classification analysis was performed on annotated transcripts using Blast2GO. As shown in Fig. 6, a total of 1,270 DEGs were screened out after a comparison between the GN and GW samples. The results showed that 1,270 DEGs that were annotated in the GO database were categorized into 52 functional groups, including the three main GO ontologies: biological processes, cellular components, and molecular functions. Among these DEGs, a large number were dominant in nine terms, including biological regulation, cellular process, metabolic process, single-organism process, cell, cell part, organelle, binding, and catalytic activity.

Then, all of the DEGs were mapped in the KEGG database to search for genes involved in the innate immune response or signaling pathways. A total of 7,000 DEGs were assigned to 250 KEGG pathways. KEGG pathway analysis showed that 36 pathways were obviously changed (P-value < 0.05) in the GW sample compared with the GN sample. Among these 36 pathways, 28 were significantly changed (P-value < 0.01), and some were related to the innate immunity response, including the insulin signaling pathway, the Wnt signaling pathway, ECM-receptor interaction, the JAK-STAT signaling pathway, cell adhesion molecules, the mRNA surveillance pathway, cytokine-cytokine receptor interaction, lysosome, adherens junction, and the Notch signaling pathway (Table 4). Because the antiviral immune mechanism of crayfish is not clear, the discovery of these above signaling pathways for DEGs will help to identify the innate immune mechanisms.

### In-depth analysis of DEGs involved in signaling pathways related to innate immunity

Based on the KEGG pathway analysis of DEGs between GN and GW, some classical signaling pathways that were related to the innate immune system were screened out, for example, the JAK-STAT signaling pathway, insulin signaling pathway, Wnt signaling pathway, mRNA surveillance pathway, and Notch signaling pathway. To date, research results regarding the antiviral immune mechanisms of crustaceans have revealed that the JAK-STAT signaling pathway is involved in the antiviral innate immune response of shrimp^23^. However, the roles of the other four signaling pathways in the crustacean antiviral immune response have not been reported.

**Figure 7:**
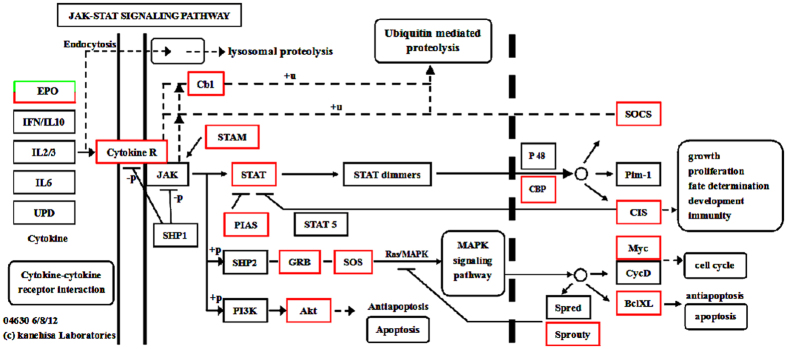
Significantly differentiated expressed genes that were identified by KEGG as involved in the JAK-STAT signaling pathway. Red boxes indicate significantly increased expression. Green boxes indicate significantly decreased expression. Black boxes indicate unchanged expression.

The JAK-STAT signaling pathway has also been implicated in the insect antiviral immune defense response, which includes three main cellular components: receptor Domeless, Janus Kinase (JAK) Hopscotch, and STAT transcription factor^24^. Moreover, the transcription of STAT in shrimp was obviously up-regulated after WSSV infection^25^, indicating that the JAK-STAT pathway might play a very important role in shrimp antivirus immunity responses. According to the transcriptome sequencing results in the present study, some unigenes were annotated in the JAK-STAT signaling pathway, and their expression levels obviously varied after WSSV infection. These results suggest that this pathway plays an important role in the crayfish antiviral innate immune response. In the present study, 21 genes were significantly differentially expressed in the JAK-STAT signaling pathway, including 19 significantly up-regulated genes and 2 significantly down-regulated genes (Fig. 7). The protein identification and concrete expression profile analysis of these 21 genes is shown in Table 5. Some important molecules involved in the classical JAK-STAT signaling pathway are included, for example, STAT, suppressor of cytokine signaling-2 like protein (SOCS), apoptosis regulator Bcl-XL, Myc protein, Ras GTP exchange factor, and Src kinase-associated phosphoprotein 2 (SKAP). Based on the transcriptome sequencing results, the gene expression levels of these abovementioned molecules were obviously up-regulated after WSSV infection (Table 5).

**Figure 8:**
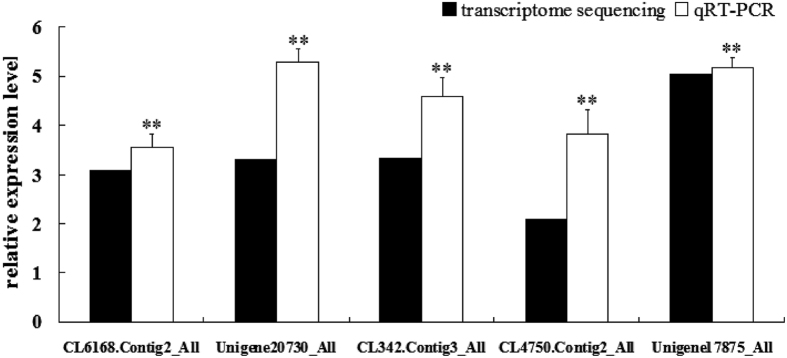
Expression profiles of the five key DEGs involved in the JAK-STAT signaling pathway after WSSV challenge. The protein identities of CL6168.Contig2_All, Unigene20730_All, CL342.Contig3_All, CL4750.Contig2_All, and Unigene17875_All were STAT, Ras GTP exchange factor, Ras GTP exchange factor, apoptosis regulator Bcl-XL, and suppressor of cytokine signaling-2 like protein, respectively. These DEGs were amplified in crayfish intestine 36 h post WSSV infection. *18S RNA* was used as an internal reference. The asterisks indicate significant differences (**P-value < 0.01) from the control.

To further ascertain the role of the JAK-STAT signaling pathway in crayfish antiviral immune responses, five key DEGs involved in the JAK-STAT signaling pathway were selected for qRT-PCR to analyze their expression profiles in crayfish intestine after WSSV infection. The protein identities of the five DEGs were STAT, Ras GTP exchange factor, Ras GTP exchange factor, apoptosis regulator Bcl-XL, and suppressor of cytokine signaling-2 like protein. The qRT-PCR results showed that these five DEGs were obviously up-regulated at 36 h post WSSV infection in crayfish intestine (Fig. 8). These results could provide new insight into crayfish antiviral immunity. To clarify the functions of this pathway, other components need to be identified, and the interaction among these components needs to be explored as soon as possible.

### Validation of transcriptome data by qRT-PCR

We selected thirteen genes that are related to the innate immunity response to evaluate their differential expression levels between GN and GW samples using qRT-PCR^26^. For these candidate genes, the qRT-PCR expression profile patterns were consistent with the RNA-Seq data (Table 6). There were similar trends of gene up/down-regulation between the qRT-PCR and data. The results illustrated that the RNA-Seq data were reliable.

## Conclusions

In this study, the *de novo*-assembled transcriptomes of crayfish intestines were analyzed, and a large amount of sequence information was obtained. The expression profiles of DEGs between the normal crayfish intestine transcriptome and that of WSSV-challenged crayfish was studied. The aim of this deep analysis of DEG functional annotation, orthologous protein clustering, and annotation of signaling pathways related to the immune system was to determine the underlying mechanisms involved in the anti-WSSV immune response in crayfish. Based on the transcriptome sequencing results in the present study, many genes and pathways related to innate immunity in crayfish intestine were regulated after WSSV challenge. It is worth noting that 7,000 DEGs were screened out after a comparative analysis between the GN and GW samples. These DEGs were mapped into 250 KEGG pathways. Among these pathways, 36 were obviously changed (P-values < 0.05) and 28 pathways were extremely significantly changed (P-values < 0.01).

To further identify the signaling pathways that were related to the crayfish antiviral immune response, five key DEGs involved in the JAK-STAT signaling pathway were selected for qRT-PCR. The results showed that all five of these DEGs were obviously up-regulated at 36 h post WSSV infection in crayfish intestine. Taken together, these results provide new insight into the crayfish antiviral immunity mechanism. In addition, these results could also provide an important theoretical basis for solving viral disease problems in crayfish breeding.

## Materials and Methods

### Preparation of crayfish tissues and immune challenge

*P. clarkii* (approximately 15–20 g) were purchased from a commercial aquaculture market in Hangzhou, Zhejiang Province, China. The collected crayfish were originally cultured in water tanks at 26–28 °C for at least 5 days and fed twice daily with artificial food throughout the experiment^27^. For WSSV infection, WSSV (3.2 × 10^7^ particles per crayfish) was injected into the abdominal segment of each crayfish^1^,^28^,^29^. Then, 36 h after challenge, the intestines were collected from no fewer than ten WSSV-challenged crayfish. These samples constituted the WSSV group (GW). The intestines were also collected from at least ten normal crayfish and frozen immediately in liquid nitrogen. These samples constituted the normal group (GN). Then, these two sets of samples were temporarily stored at −80 °C for total RNA extraction^27^.

### RNA isolation and Illumina sequencing

The two sets (GN and GW) of intestine tissue samples that had been frozen in liquid nitrogen were delivered to the Beijing Genomics Institute-Shenzhen (BGI, Shenzhen, China) for total RNA extraction. In brief, the total RNA from the crayfish intestines was extracted with TRIzol reagent in accordance with the manufacturer’s protocol (Invitrogen, USA). The quality of the RNA sample after treatment with DNase I (Invitrogen) was examined before continuing to the subsequent procedures, including mRNA purification, cDNA library construction and transcriptome sequencing. Approximately 5 μg of DNase-treated total RNA was used to construct a cDNA library following the protocols of the Illumina TruSeq RNA Sample Preparation Kit (Illumina, USA). After necessary quantification and qualification, the library was sequenced using an Illumina HiSeq™ 2000 instrument with 100 bp paired-end (PE) reads for GN and GW.

### Transcriptome *de novo* assembly and analysis

Transcriptome *de novo* assembly for the two intestine sample (GN and GW) sets was carried out by the RNA-Seq *de novo* assembly program Trinity^30^. In brief, the raw reads that were generated by the Illumina HiSeq™ 2000 sequencer were originally trimmed by removing the adapter sequences. After the low-quality reads with quality scores of less than 20 and short reads with lengths of less than 10 bp were removed, high-quality clean reads were obtained to perform transcriptome *de novo* assembly using Trinity software with the default parameters. Generally, there were three steps, including Inchworm, Chrysalis and Butterfly^31^. In the first step, high-quality clean reads were processed by Inchworm to form longer fragments, which were called contigs. Then, these contigs were connected by Chrysalis to obtain unigenes that could not be extended on either end. These unigenes resulted in de Bruijn graphs. Finally, the de Bruijn graphs were processed by Butterfly to obtain transcripts^32^.

### Transcriptome annotation and gene ontology analysis

After transcriptome *de novo* assembly, the transcripts were used for annotation, including protein functional annotation, COG functional annotation, GO functional annotation, and pathway annotation. These processes are based on sequence similarity with known genes. In detail, the assembled contigs were annotated with sequences available in the NCBI database using the BLASTx and BLASTn algorithms^33^. The unigenes were aligned by a BLASTx search against the protein databases of NCBI, including Nr, Swiss-Prot, KEGG, and COG^34^. Meanwhile, none of the BLASTx hits were aligned by a BLASTn search against the NCBI Nt database. All of the above alignments were executed to establish the homology of sequences with known genes (with a cutoff E-value ≤ 10^−5^)^35^. Then, the best alignment results were used to determine the sequence direction and protein-coding-region prediction (CDS) of the unigenes. Functional annotation was executed with GO terms (www.geneontology.org) that were analyzed using Blast2GO software (http://www.blast2go.com/b2ghome)^36^. Based on the KEGG database, the complex biological behavior of the genes was analyzed through pathway annotation.

### Identification of differentially expressed genes

To acquire the expression profiles for transcripts in crayfish intestines, cleaned reads were first mapped to all of the transcripts using Bowtie software^37^. Then, DEGs were obtained based on the number of fragments per kilobase of exon per million fragments mapped (FPKM) of the genes, followed by a False Discovery Rate (FDR) control to correct for the P-value^38^. DEGs were identified using EDGER software (empirical analysis of digital gene expression data in R)^39^. For this analysis, the filtering threshold was set as an FDR control of 0.5. Lastly, FDR ≤ 0.001 and the absolute value of log2Ratio ≥ 1 were used as the filtering thresholds to determine the significance of the differentially expressed genes^40^. The justification for using |log2Ratio| ≥ 1 as the filtering threshold was to reduce the statistical workload while obtaining more meaningful differentially expressed genes. Using this method, the differentially expressed genes were identified between GW and GN through a comparative analysis of the above data.

### Quantitative real-time PCR validation

Quantitative real-time PCR (qRT-PCR) methods were used to determine the RNA levels for fifteen selected genes that were related to the innate immune response^41^. For qRT-PCR analysis, cDNA templates from the two intestine sample (GN and GW) sets were diluted 20-fold in nuclease-free water and were used as templates for PCR. Gene-specific primer sequences were carefully designed using Primer Premier 6 software based on the sequence of each gene that was identified from the transcriptome library^42^. The specific primers, namely *Pc-18 S RNA*-qRT-F (5′-tct tct tag agg gat tag cgg-3′) and *Pc-18 S RNA*-qRT-R (5′-aag ggg att gaa cgg gtt a-3′), were used to amplify the *18S RNA* gene as the inner control. qRT-PCR was performed following the manufacturer’s instructions for SYBR Premix Ex Taq (Takara, Japan) using a real-time thermal cycler (Bio-Rad, USA) in a total volume of 10 μl containing 5 μl of 2× Premix Ex Taq, 1 μl of the 1:20 diluted cDNA, and 2 μl (1 μM) each of the forward and reverse primers. The amplification procedure comprised an initial denaturation step at 95 °C for 3 min, followed by 40 cycles of 95 °C for 15 s and 59 °C for 40 s and melting from 65 °C to 95 °C. Three parallel experiments were performed to improve the integrity of the work^43^. Furthermore, the differentially expressed levels of the target genes (between the GN and GW samples) were calculated by the 2^−ΔΔCT^ analysis method as described in a previous study^44^. The obtained data were subjected to statistical analysis, followed by an unpaired sample *t*-test. A significant difference was accepted at a P-value < 0.05. An extremely significant difference was accepted at P < 0.01.

## Additional Information

**How to cite this article**: Du, Z. *et al*. In-depth comparative transcriptome analysis of intestines of red swamp crayfish, *Procambarus clarkii*, infected with WSSV. *Sci. Rep.* **6**, 26780; doi: 10.1038/srep26780 (2016).

## Figures and Tables

**Table 1:** Summary of the Illumina sequencing output for the GN and GW samples.

Sample	Total Raw Reads	Total Clean Reads	Total Clean Nucleotides (nt)	Q20 percentage	N percentage	GC percentage
GN-intestine	46,945,132	44,600,142	4,460,014,200	98.05%	0.00%	40.45%
GW-intestine	49,574,674	47,790,746	4,779,074,600	97.96%	0.00%	41.58%

**Table 2:** Summary of the assembly analysis of the GN and GW samples.

Dataset name	Normal group (GN)		WSSV group (GW)	
	Contigs	Unigenes	Contigs	Unigenes
Total Number	125,394	70,791	148,983	83,043
Total Length (nt)	44,957,246	44,400,732	54,762,609	55,831,345
Mean Length (nt)	359	627	368	672
N50	701	1258	769	1,456
Total Consensus sequences	–	70,791	–	83,043
Distinct Clusters	–	7,352	–	9,686
Distinct Singletons	–	63,439	–	73,357

**Table 3:** The top 50 statistically significant KEGG classifications.

No.	Pathway	Pathway definition	Number of sequences
1	path: ko01100	Metabolic pathways	3371 (13.33%)
2	path: ko05146	Amoebiasis	1148 (4.54%)
3	path: ko05110	Vibrio cholerae infection	1092 (4.32%)
4	path: ko05016	Huntington’s disease	973 (3.85%)
5	path: ko04810	Regulation of actin cytoskeleton	950 (3.76%)
6	path: ko04510	Focal adhesion	910 (3.6%)
7	path: ko03040	Spliceosome	894 (3.53%)
8	path: ko05200	Pathways in cancer	879 (3.48%)
9	path: ko05169	Epstein-Barr virus infection	860 (3.4%)
10	path: ko03013	RNA transport	847 (3.35%)
11	path: ko00230	Purine metabolism	781 (3.09%)
12	path: ko04145	Phagosome	643 (2.54%)
13	path: ko04144	Endocytosis	638 (2.52%)
14	path: ko04270	Vascular smooth muscle contraction	633 (2.5%)
15	path: ko03010	Ribosome	632 (2.5%)
16	path: ko04530	Tight junction	616 (2.44%)
17	path: ko00240	Pyrimidine metabolism	616 (2.44%)
18	path: ko04142	Lysosome	610 (2.41%)
19	path: ko04141	Protein processing in endoplasmic reticulum	607 (2.4%)
20	path: ko05166	HTLV-I infection	596 (2.36%)
21	path: ko05168	Herpes simplex infection	593 (2.34%)
22	path: ko05132	Salmonella infection	576 (2.28%)
23	path: ko03015	mRNA surveillance pathway	563 (2.23%)
24	path: ko04120	Ubiquitin mediated proteolysis	557 (2.2%)
25	path: ko04010	MAPK signaling pathway	542 (2.14%)
26	path: ko04020	Calcium signaling pathway	538 (2.13%)
27	path: ko05414	Dilated cardiomyopathy	524 (2.07%)
28	path: ko05164	Influenza A	517 (2.04%)
29	path: ko05410	Hypertrophic cardiomyopathy (HCM)	495 (1.96%)
30	path: ko04970	Salivary secretion	493 (1.95%)
31	path: ko04976	Bile secretion	491 (1.94%)
32	path: ko05202	Transcriptional misregulation in cancer	484 (1.91%)
33	path: ko05010	Alzheimer’s disease	482 (1.91%)
34	path: ko05130	Pathogenic Escherichia coli infection	467 (1.85%)
35	path: ko04520	Adherens junction	467 (1.85%)
36	path: ko05152	Tuberculosis	464 (1.83%)
37	path: ko04910	Insulin signaling pathway	464 (1.83%)
38	path: ko04310	Wnt signaling pathway	438 (1.73%)
39	path: ko04110	Cell cycle	431 (1.7%)
40	path: ko04114	Oocyte meiosis	428 (1.69%)
41	path: ko04062	Chemokine signaling pathway	428 (1.69%)
42	path: ko04512	ECM-receptor interaction	424 (1.68%)
43	path: ko03020	RNA polymerase	423 (1.67%)
44	path: ko05100	Bacterial invasion of epithelial cells	411 (1.63%)
45	path: ko04971	Gastric acid secretion	410 (1.62%)
46	path: ko02010	ABC transporters	407 (1.61%)
47	path: ko03008	Ribosome biogenesis in eukaryotes	391 (1.55%)
48	path: ko05131	Shigellosis	374 (1.48%)
49	path: ko04972	Pancreatic secretion	372 (1.47%)
50	path: ko04666	Fc gamma R-mediated phagocytosis	367 (1.45%)

**Table 4:** Top 36 differentially expressed pathways between the GW and GN samples.

No.	Pathway	Number of DEGs	P-value	Pathway ID
1	Glycolysis/Gluconeogenesis	23 (0.79%)	2.57E-23	ko00010
2	Insulin signaling pathway	46 (1.58%)	6.94E-11	ko04910
3	Wnt signaling pathway	40 (1.38%)	8.07E-10	ko04310
4	ECM-receptor interaction	77 (2.65%)	3.82E-08	ko04512
5	JAK-STAT signaling pathway	21 (0.72%)	8.36E-07	ko04630
6	Pancreatic secretion	45 (1.55%)	2.72E-06	ko04972
7	Cell adhesion molecules (CAMs)	33 (1.13%)	3.20E-05	ko04514
8	mRNA surveillance pathway	90 (3.09%)	7.51E-05	ko03015
9	Arrhythmogenic right ventricular cardiomyopathy	38 (1.31%)	0.000101	ko05412
10	Gap junction	24 (0.83%)	0.000149	ko04540
11	Basal transcription factors	23 (0.79%)	0.000154	ko03022
12	Cytokine-cytokine receptor interaction	15 (0.52%)	0.000166	ko04060
13	Viral myocarditis	61 (2.1%)	0.000272	ko05416
14	Lysosome	54 (1.86%)	0.000495	ko04142
15	RNA degradation	25 (0.86%)	0.000764	ko03018
16	Adherens junction	52 (1.79%)	0.001167	ko04520
17	Natural killer cell mediated cytotoxicity	11 (0.38%)	0.001206	ko04650
18	Arginine and proline metabolism	17 (0.58%)	0.001484	ko00330
19	Drug metabolism - cytochrome P450	13 (0.45%)	0.001743	ko00982
20	Notch signaling pathway	28 (0.96%)	0.001809	ko04330
21	Huntington’s disease	82 (2.82%)	0.003241	ko05016
22	Cocaine addiction	10 (0.34%)	0.004265	ko05030
23	Dilated cardiomyopathy	87 (2.99%)	0.004299	ko05414
24	Shigellosis	54 (1.86%)	0.005942	ko05131
25	Bile secretion	59 (2.03%)	0.007031	ko04976
26	Alanine, aspartate and glutamate metabolism	8 (0.28%)	0.007314	ko00250
27	Riboflavin metabolism	6 (0.21%)	0.007432	ko00740
28	Vascular smooth muscle contraction	125 (4.3%)	0.009123	ko04270
29	Ribosome	8 (0.28%)	0.012305	ko03010
30	Glycosaminoglycan biosynthesis-heparan sulfate	33 (1.13%)	0.014108	ko00534
31	Phototransduction - fly	18 (0.62%)	0.014165	ko04745
32	Mineral absorption	16 (0.55%)	0.016715	ko04978
33	Pertussis	24 (0.83%)	0.017989	ko05133
34	Porphyrin and chlorophyll metabolism	6 (0.21%)	0.035561	ko00860
35	Circadian rhythm - mammal	3 (0.1%)	0.044674	ko04710
36	Salmonella infection	83 (2.85%)	0.046557	ko05132

**Table 5:** Identification and expression profile analysis of 21 DEGs involved in the JAK-STAT signaling pathway.

No.	Gene name	Protein identity	Fold variation (GW/GN) in transcriptome sequencing
1	CL3474.Contig3_All	DNA polymerase sigma subunit	11.80 (up)
2	Unigene8920_All	Keratin-associated protein	6.96 (up)
3	Unigene17875_All	Suppressor of cytokine signaling-2 like protein	5.03 (up)
4	Unigene12483_All	E3 ubiquitin-protein ligase	4.11 (up)
5	CL152.Contig1_All	Sporozoite surface protein	3.53 (up)
6	CL342.Contig3_All	Src kinase-associated phosphoprotein 2	3.34 (up)
7	Unigene20730_All	Ras GTP exchange factor	3.32 (up)
8	Unigene2560_All	Cytokine signaling-2	3.29 (up)
9	Unigene34040_All	Akt	3.25 (up)
10	CL6168.Contig2_All	STAT	3.07 (up)
11	CL1132.Contig6_All	Myc protein	2.66 (up)
12	Unigene22783_All	Protein kinase C	2.51 (up)
13	Unigene25712_All	Mediator of RNA polymerase II	2.48 (up)
14	Unigene33180_All	Peroxidasin homolog	2.38 (up)
15	CL2846.Contig1_All	Ran-binding protein 9	2.23 (up)
16	CL6025.Contig2_All	Zinc finger MIZ domain-containing protein	2.19 (up)
17	CL4750.Contig2_All	Apoptosis regulator Bcl-XL	2.10 (up)
18	Unigene41496_All	CREB-binding protein	2.06 (up)
19	Unigene24833_All	Integrator complex subunit 6	2.03 (up)
20	Unigene12332_All	Peroxinectin	2.58 (down)
21	CL6023.Contig1_All	Chorion peroxidase	2.16 (down)

**Table 6:** Comparison of the relative fold change of the RNA-Seq and qRT-PCR results between the GW and GN samples.

No.	Gene name	Protein identity	Fold variation (GW/GN)	
			transcriptome	qRT-PCR
1	Unigene13115_All	Caspase	10.67 (up)	6.55 (up)
2	CL1181.Contig5_All	Integrin	7.70 (up)	5.39 (up)
3	CL3734.Contig4_All	Serine proteinase inhibitor	6.78 (up)	4.24 (up)
4	Unigene1712_All	Single WAP domain-containing protein	6.62 (up)	5.17 (up)
5	CL1460.Contig3_All	Cellular apoptosis susceptibility protein	5.54 (up)	6.88 (up)
6	Unigene8956_All	Toll like receptor	5.28 (up)	3.27 (up)
7	CL2879.Contig2_All	I-type lysozyme-like protein	4.73 (up)	4.18 (up)
8	CL2506.Contig2_All	C-type lectin	4.64 (up)	2.59 (up)
9	Unigene8963_All	Dicer-1	3.71 (up)	5.83 (up)
10	Unigene42500_All	Anti-lipopolysaccharide factor	2.57 (up)	5.76 (up)
11	Unigene10305_All	Glutathione peroxidase	0.18 (down)	0.32 (down)
12	Unigene12675_All	Glutathione S-transferase T2	0.26 (down)	0.29 (down)
13	Unigene38817_All	Serine proteinase-like 2a	0.45 (down)	0.37 (down)
